# Gestational Diabetes Mellitus Changes Human Colostrum Immune Composition

**DOI:** 10.3389/fimmu.2022.910807

**Published:** 2022-06-20

**Authors:** Ana Carolina de Sena Avellar, Mariana Naves Oliveira, Felipe Caixeta, Rafaela Cristina Vieira e Souza, Andréa Teixeira, Ana Maria Caetano Faria, Gabriela Silveira-Nunes, Elaine Spezialli Faria, Tatiani Uceli Maioli

**Affiliations:** ^1^Departamento de Nutrição, Programa de Pós-graduação em Nutrição e Saúde, Universidade Federal de Minas Gerais, Belo Horizonte, Brazil; ^2^Hospital Sofia Feldman, Belo Horizonte, Brazil; ^3^Programa Interunidades de Pós-Graduação em Bioinformática, Universidade Federal de Minas Gerais, Belo Horizonte, Brazil; ^4^Instituto de Pesquisa Rene Rachou, Fundação Oswaldo Cruz, Belo Horizonte, Brazil; ^5^Departamento de Bioquímica e Imunologia, Universidade Federal de Minas Gerais, Belo Horizonte, Brazil; ^6^Universidade Federal de Juiz de Fora, Campus Governador Valadares, Belo Horizonte, Brazil

**Keywords:** gestational diabetes mellitus, colostrum, immune system, cytokines, chemokines, growth factors

## Abstract

Breast milk is considered a complete food for babies. Up to 7 days postpartum, it is known as colostrum, rich in immunological compounds, responsible for providing nutrition and ensuring immune protection. However, some maternal factors, such as gestational diabetes mellitus (GDM), can change the concentration of bioactive compounds present in the colostrum and may affect the development of the newborn’s immune system. The effect of GDM on colostrum cytokine, chemokine, and growth factors is not well described. Thus, the present study evaluated whether the occurrence of GDM changes the concentration of biomarkers in the colostrum. A cross-sectional study was carried out on postpartum women who had healthy pregnancies and women who had been diagnosed with GDM. A sample of colostrum was collected for Luminex analysis. Our results showed that GDM mothers had higher secretion of cytokines and chemokines in the colostrum, with a higher concentration of IFN-g, IL-6, and IL-15, and a lower concentration of IL-1ra. Among growth factors, we identified a decreased concentration of GM-CSF in the colostrum of GDM mothers. Thus, the data obtained support the idea that the disease leads to immune alterations in the colostrum.

## Introduction

Human breast milk is a complex fluid that is considered as the ideal food to support newborns’ growth until 6 months and promote their adaptation to the environment (1, 2). Besides nutritional molecules, such as lipids, carbohydrates, and proteins, human breast milk is composed of immune cells, bioactive molecules such as immunoglobulins, lactoferrin, lysozyme, cytokines, chemokines, and growth factors. These molecules are essential to promote the correct formation of the newborn’s immune system and support organ development (3, 4). In addition, these molecules are present especially in the colostrum, providing protection for newborns against infections (5).

Colostrum is the first milk to be secreted from the breast and lasts until approximately the 7th day of the newborn’s life, and is also characterized as the most potent natural immune mediator (6). Among the functions of the colostrum are the provision of passive and active immune defense, the modulation of the immune response in the newborn, the development of the intestinal microbiota, and the growth and repair of various tissues, including the intestinal mucosa (7, 8). In addition, the colostrum has high levels of immunoglobulins, cytokines, and immune cells compared to mature milk (9, 10). Furthermore, the colostrum can protect the infant from allergic and chronic diseases and protect the infant from hypertension, type 2 diabetes, and excess weight (11, 12).

Some factors can influence colostrum composition, such as genetics, environment and geographical location, food consumption, lactation stage, and maternal nutritional status and health (13).

Gestational diabetes mellitus (GDM) is characterized by carbohydrate intolerance during pregnancy, and it starts mainly in the 20th week of the gestational period. The prevalence of this condition is increasing due to the increased incidence of obesity, late pregnancy, and lack of physical activity. Thus, this condition is present in 3% to 25% of pregnant women (14), and the consequence to the newborn is metabolic alteration, steatosis, excess weight, and less access to breast milk (15). Also, women with GDM present alterations in their immune profile, increasing the production of inflammatory cytokines and increasing the proportion of Th17 cells (16, 17). This inflammatory environment can be mirrored in the colostrum, bringing consequences to the infant.

Even though some consequences of GDM have already been described, it is still unclear how this disease alters the profile of cytokines and chemokines present in breast milk, and whether these changes can compromise the health of the child. Thus, in the present study, we aimed to evaluate the profile of cytokines and chemokines present in the colostrum of women who presented with GDM compared to those with the usual risk during pregnancy.

## Methods

### Design and Sample

A cross-sectional study was carried out with the sample size calculated using the formula for case–control studies with a continuous outcome variable; considering the standard deviation and expected difference in the concentration of cytokines in breast milk between the groups, information obtained in a previous study (18), and a statistical power of 95% and a significance level of 5%, a minimum of five participants in each group was estimated.

The sample was composed of 25 colostrum donors enrolled at the Hospital Risoleta Tolentino Neves in 2018–2019. The present study was initially presented to women who voluntarily participated in accordance with the guidelines from the Institutional Committee for Ethics in Research of the Hospital Risoleta Tolentino Neves (number 13/2015).

The exclusion criteria for the sample were postpartum women under 18 years of age, women who had infectious diseases during pregnancy or other types of diabetes, use of antibiotics during pregnancy, gestational age at delivery less than 37 weeks, twin pregnancies, and those who had an absence of at least 2 ml of colostrum extraction.

The sample consisted of 25 women subdivided into two groups. For the control group (*n* = 14), puerperal women in the immediate postpartum period with normal-risk pregnancy with a full-term pregnancy, single pregnancy, without previous and present diseases, and with normal weight were considered. The other group (*n* = 11) consisted of postpartum women who were diagnosed with GDM during pregnancy according to the information obtained in the medical records of the abnormal OGTT. The GDM was diagnosed by the OGTT after the 20th week of pregnancy. In addition to colostrum collection, the puerperal women completed a structured questionnaire that included sociodemographic and anthropometric data on the pre- and post-gestational state and the presence of GDM during pregnancy. The variables evaluated in the study were as follows: pre-pregnancy and postpartum nutritional status, through weight, height, and BMI calculation, weight gain during pregnancy, and concentration of cytokines/chemokines in the colostrum.

### Colostrum Preparation and Bioactive Compound Detection

The colostrum samples were collected manually by the donor *via* sterile plastic tubes between 12 and 48 h postpartum. Colostrum was collected between feeding intervals, during daytime (between 13:00 and 16:00 h), and a minimum of 2 ml was required for all women. The samples were placed in a 15-ml tube kept on ice until processing. Then, the samples were centrifuged at 500*g* for 10 min at 4°C. After the procedure, the samples clearly showed a partition into three phases: the cream content at the top was discarded; the supernatant was removed, aliquoted in a 1.5-ml conical tube, and stored in a freezer at −80°C for the next steps of the analysis; and in the third phase, the cell content remained, which was determined to quantify the total number of cells for further studies.

The supernatants were used in the Luminex assay to detect cytokine and chemokine concentration. The kit was purchased from Bio-Rad Laboratories (Bio-Plex^®^ Pro Human Cytokine Standard). Subsequently, the Luminex equipment performed fluorescence reading (BioPlex 200, Bio-Rad).

Colostrum samples were quickly thawed in a water bath at 37°C and then homogenized by vortexing for 5 s. Due to the presence of debris in the sample after thawing, filtration was necessary to prevent particles from influencing the detection and quantification of cytokines in the fluid. Afterwards, the samples were again homogenized by vortexing for 5 s and centrifuged at 14,000*g* for 5 min at room temperature; 50 μl of the sample was added to the Luminex assay plate containing 50 μl of the bead pool, washed twice with 100 μl of wash buffer, and incubated overnight at 4°C with shaking. The following day, 25 μl of the detection antibody was added to each well, and the samples were incubated for 30 min under agitation at room temperature and protected from light. Samples were washed with 100 μl of wash buffer. Then, 50 μl of the conjugate (Streptavidin) was added to each well and the samples were again incubated for 30 min under agitation at room temperature and protected from light. They have been washed again with 100 μl of wash buffer, resuspended with 125 μl of assay buffer, and homogenized for 10 min. Finally, the samples were acquired on the Bio-Plex 200 equipment (Bio-Rad) using the Luminex PONENT software version 3.1.

Subsequently, the data were analyzed using Bioplex™ software (Bio-Rad). The panel of cytokines, chemokines, and growth factors detected by the kit consisted of the following: eotaxin (CCL11), IL-1ra, IL-1β, IL-2, IL-4, IL-5, IL-6, IL-7, IL-8, IL-9, IL-10, IL-12, IL-13, IL-15, IL-17, IP-10, MCP-1 (CCL2), MIP-1α (CCL3), MIP-1β (CCL4), RANTES (CCL5), TNF-α, IFN-γ, VEGF, FGF-basic, GM-CSF, PDGF, and G-CSF. The concentrations of cytokines and chemokines were calculated according to the standard curve for each one individually. For samples with a reading below the detection level, the value 0 was used in the analysis.

### Statistical Analysis

The questionnaire data were tabulated using the Excel 2007 program, and then univariate and multivariate descriptive analyses were performed using the Statistical Package for the Social Sciences (SPSS) version 19.0 and the Minitab version 17 program. The Kolmogorov–Smirnov test was performed to evaluate the normality or non-normality of the variables, central tendency (means and medians), and dispersion (standard deviation, minimum, and maximum values). Consequently, the chi-square correlation tests were applied to estimate the association between two qualitative variables. The Student’s *t*-test was used to compare two independent means and the Mann–Whitney *U* test was used to compare two independent medians. Then, the Bonferroni correction was applied to identify the statistical significance of some variables. For all tests performed, significance with *p* < 0.05 was considered as a statistically significant difference.

### Radar Plots

Radar graphics were performed to identify the frequency of samples that were considered low (<global median) and high (≥global median) producers of cytokine and chemokine. The global median of each component was used as the cutoff point. Each axis of the graphs represents the percentage (%) of volunteers who have a high production of cytokines and chemokines in the colostrum. The values from each axis can be connected to form a central polygonal area representing the overall Th1, regulatory, and Th2 balance. The increase or decrease in the central polygonal area reflects a greater or lesser contribution of the Th1, regulatory, and Th2 balance (19, 20).

### Spearman Correlation Analysis

Spearman’s correlation graphs were used to assess the association between cytokines and numerical variables to try to estimate the strength of this relationship. In correlated data, a change in the magnitude of one cytokine is associated with a change in the magnitude of another variable, either in the same direction (positive correlation) or in the opposite direction (negative correlation). Correlation coefficients range from −1 to +1, where 0 indicates no association, while the correlation becomes more positive as it approaches +1 and more negative as it approaches −1. A confidence interval was not made for a definitive conclusion on the strength of the relationship between the variables. Therefore, for the present study, only one correlation was evaluated according to the intensity of the colors. It was not possible to perform a regression analysis of the data due to the small sample size, and after some attempts, the regression model would have weak values to explain the outcome.

## Results

Our sample is composed of 25 women who met all the inclusion criteria. These participants were divided into two groups: a group considered to have a normal-risk pregnancy (56%) (healthy) and a group who had gestational diabetes (44%) (GDM). Some social data were evaluated, and age was an uncategorized variable; the youngest was a participant aged 18 years, and the mean and standard deviation of this variable were calculated for the healthy group (24.86 ± 5.26) and the GDM group (30.09 ± 6.25). Regarding pre-gestational nutritional status, all participants in the healthy group had normal BMI, while 54.5% of women with GDM were obese. Considering the IOM recommendations for weight gain, it is possible to observe higher weight gain during the gestational period in the GDM group. As described in **Table 1**, there was no difference in the gestational age at delivery between groups.

**Table 1:** Maternal characteristics.

Maternal Characteristics	Healthy	GDM
**Age (years)^Ø^**	30.09 ± 6.25	24.86 ± 5.26
**Pre-gestational nutritional status^§^**		
Normal	14 (100%)^a^	1 (9.1%)^b^
Overweight	0^a^	4 (36.4%)^b^
Obese	0^a^	6 (54.5%)^b^
**Gestational weight gain**		
Insufficient	7 (50.0%)^a^	2 (18.1%)^b^
Adequate	6 (42.9%)	4 (36.4%)
Excessive	1 (7.1%)^a^	5 (45.5%)^b^
**Child weight at birth ^Ø^**	(3,233.18 ± 394.40)	(3,132.50 ± 388.40)
**Gestational age at delivery^¥^(weeks)**	38.57 ± 1.65	39.09 ± 1.51

^§^Chi-square test; ^Ø^Simple Student’s t-test; ^¥^Mann–Whitney test. Frequencies followed by different letters between the categories represent a statistically significant difference between groups (p < 0.05).

The primary purpose of this work was to evaluate whether GDM could influence the bioactive factor present in the human colostrum. The **Supplementary Table** indicates all bioactive factors measured.

**Figure 1:**
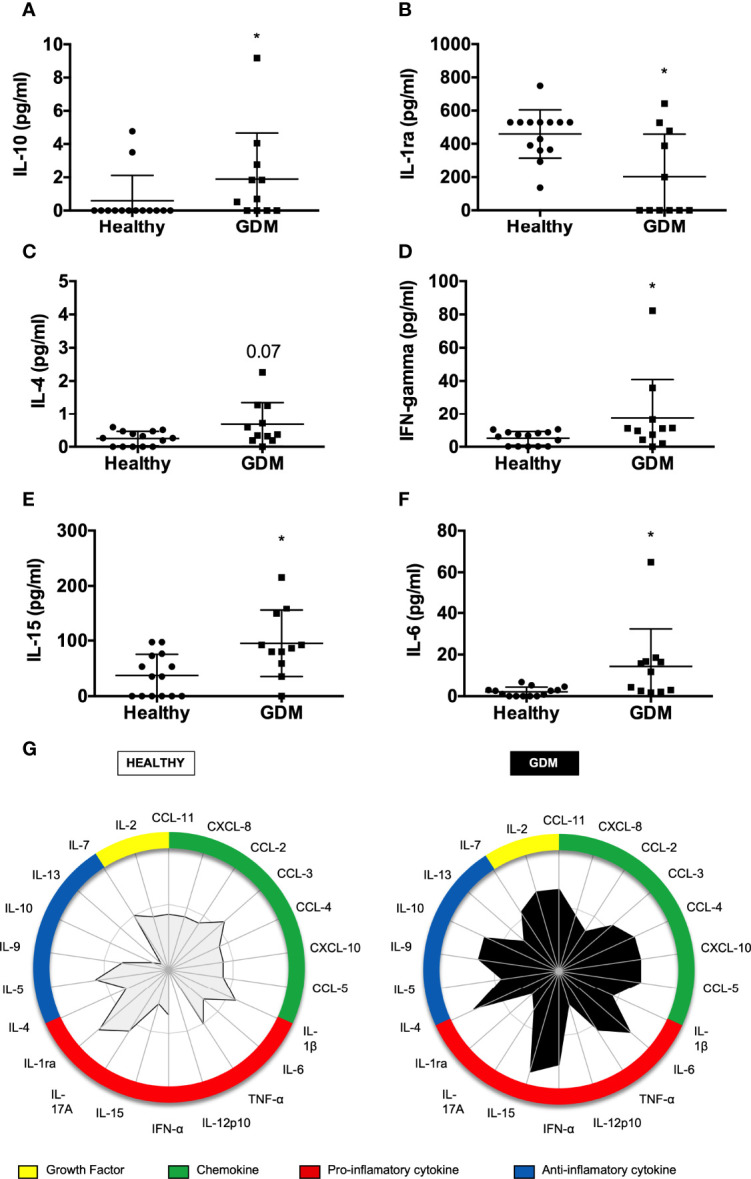
Comparison of cytokine levels in colostrum between healthy and GDM mothers. The concentrations of cytokines and chemokines were measured in colostrum by Luminex. The circles represent women classified as having a usual gestational risk. The squares represent women with GDM. A total of 25 samples were included in the analyses. Each geometric figure represents a participant. **(A)** IL-10, **(B)** IL-1ra, **(C)** IL-4, **(D)** IFN-γ, **(E)** IL-5, **(F)** IL-6. *p < 0.05. **(G)** Global profile of cytokines/chemokines in colostrum of postpartum women, according to risk classification during pregnancy. The radar graph shows the frequency of high producers of pro-inflammatory, anti-inflammatory, and chemokines levels, and the contribution of each in the different categorizations (habitual risk and GDM).

The results showed interesting differences between groups. We observed a higher concentration of IL-10, IFN-γ, IL-15, and IL-6 in the GDM mothers’ colostrum (**Figures 1A, D–F**), and we observed a decreased secretion of IL-1ra (**Figure 1B**). Regarding IL-4 (**Figure 1C**) GDM mothers' colostrum tended to have a higher concentration. To further increase the secretion of cytokines and chemokines in the human colostrum, individual samples were stratified in either low or high cytokine producers taking the global median of the cytokine index as the cutoff (**Figure 1G**), as previously reported by our group (20). When considering the frequency of high cytokine and chemokine producers, a relevant secretion was identified in the colostrum of the GDM group observed in the central polygonal area. Increasing or decreasing the central polygon areas reflects the greater or lesser contribution of the balance of these cytokines. Thus, it is observed that women with GDM have a higher frequency of production of inflammatory cytokines and cytokines in the colostrum.

**Figure 2:**
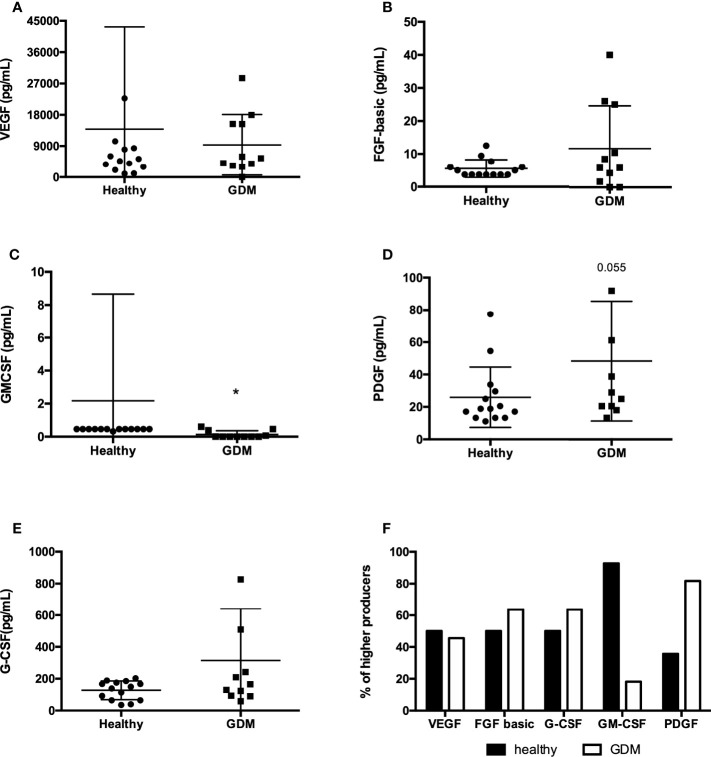
Concentration of growth factors in colostrum comparing healthy mothers with GDM. The concentrations of growth factors were measured in colostrum by Luminex. The circles represent women classified as having a usual gestational risk. The squares represent women with GDM. A total of 25 samples were included in the analyses. Each geometric figure represents a participant. **(A)** VEF, **(B)** FGF-basic, **(C)** GM-CSF, **(D)** PDGF, **(E)** G-CSF; *p < 0.05. **(F)** Frequency of high producers of growth factors in colostrum.

The human colostrum contains numerous growth factors that have wide-ranging effects on the maturation of an infant’s intestinal mucosa. We evaluated the presence of five growth factors in the human colostrum from mothers with GDM. Comparing healthy mothers to mothers with GDM, we observed a statistically significant difference in GM-CSF presence in the colostrum. GDM decreases the presence of this growth factor (**Figure 2C**). We did not observe the difference in the other growth factors examined (**Figures 2A, B, E**), but the PDGF tended to be more secreted in the GDM’s colostrum (Figure 2D). Using the same approach of higher producers for growth factor analysis (**Figure 2F**), we can note that in mothers with GDM, fewer samples had a high concentration of GM-CSF, while more samples had a high PDGF concentration compared to healthy ones.

**Figure 3:**
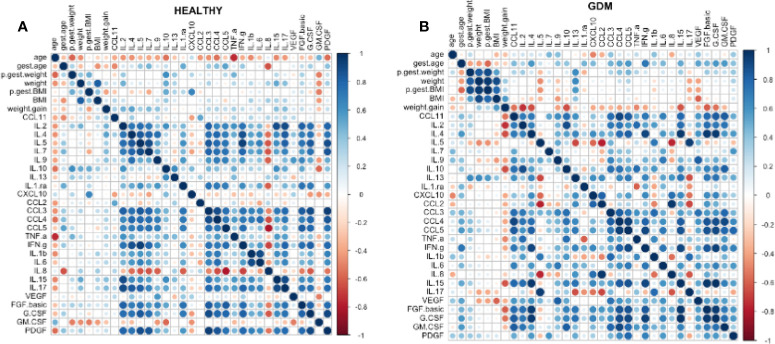
Spearman’s correlation was performed to verify the correlation between the 27 bioactive factors measured in the colostrum and the numerical variables. In the healthy group, dot colors are less intense, and we observed a more positive correlation between the bioactive factors themselves **(A)**. A more significant correlation between the variables was observed mainly in the group of women who presented with GDM **(B)**, through the intensity of the colors (more intense blue = stronger correlation). In addition, there is a negative correlation (more intense red = stronger negative correlation) between weight gain and the biomarkers.

Spearman’s correlation was performed to verify the correlation between the 27 bioactive factors measured in the colostrum and the numerical variables. A more significant correlation between the variables was observed mainly in the group of women who presented with GDM (**Figure 3B**), through the intensity of the colors (more intense blue = stronger correlation). In addition, there is a negative correlation (more intense red = stronger negative correlation) between weight gain and the biomarkers. In the healthy group, colors are less intense, and we observed a more positive correlation between the bioactive factors themselves (**Figure 3A**).

## Discussion

The colostrum is the first milk secreted by breast glands after birth, and aside from its nutritional benefits, the colostrum is essential to providing passive immunity to the newborn and contributes to the maturation of the immature and recently exposed immune system (21, 22). The colostrum is rich in proteins, and among the proteins, bioactive proteins such as cytokines, hormones, chemokines, and growth factors stand out. However, because these compounds are complex, there are many variables that could affect the evaluation of its concentration, such as variability according to the gestational age at delivery (23, 24), ethnicity (25, 26) (27), the time of collection (28), and diet (29). Studies also suggest that maternal nutritional status also compromises the composition of bioactive proteins in the colostrum (11, 30), but little is known about the effect of gestational diseases such as gestational hypertension and diabetes on these compounds.

The newborn is transferred from a sterile environment to an external environment colonized by microorganisms and in contact with different antigens (5). However, the colostrum is rich in cytokines, cells, and antibodies that will help in the maturation of the child’s immune system from that moment (6, 31). How these cytokines and chemokines survive passing through the baby’s stomach remains unknown, but some studies suggest that they can be sequestered until they reach the intestine (32). It is also believed that there is a difficulty in evaluating the role of each cytokine in the baby’s immune system. Since the concentrations of these compounds vary greatly, what is known is that their ingestion through breast milk influences the maturation and the development of cells in this system in newborns (33, 34).

The immune composition of breast milk changes in the presence of GDM, a disease recognized as a global pandemic characterized not only by increased insulin resistance and glucose intolerance but also by a state of low-grade systemic inflammation and dysregulation of the immune system (12, 35), which induces an imbalance between Th1 and Th2 cells favoring inflammatory responses. Furthermore, due to changes in glucose metabolism, the components of milk that these women produce are also altered (16). Therefore, in this brief report, we have shown that GDM can cause alteration in the secretion of a bioactive factor in the human colostrum, with an increased concentration of inflammatory cytokines and a decrease in the GM-CSF.

GDM is positively correlated with pre-gestational obesity and excessive weight gain during pregnancy, which may be one of the reasons for the inflammatory profile observed in pregnant women with GDM. In our sample, 90.9% of women with GDM were overweight or obese before pregnancy, thus confirming the findings of previous studies that overweight or obese women before pregnancy are at increased risk of developing GDM (36, 37). The altered immune profile observed in the blood of GDM women also impacts the composition of breast milk. Colostrum and breast milk from GDM mothers present decreased levels of antibodies and less monocyte phagocytic capacity (35). However, another study showed no difference in the mature milk composition regarding cytokines and hormones comparing healthy to GDM mothers (13).

We have analyzed 27 different molecules secreted in the colostrum upon GDM and found a difference in the cytokine profile comparing the colostrum from GDM mothers to healthy ones. According to the data, women with GDM showed higher secretion of inflammatory cytokines such as IL-15, IL-6, and IFN-γ. The analysis of the global profile of cytokines and chemokines represented by the radar graphs compares the contribution of all cytokines and chemokines simultaneously. It allows the assessment of a greater, relative contribution of each cytokine compared to the others, according to the categorization of the groups. After examining these graphs, it was possible to observe that in the group of women with GDM, most cytokines and chemokines, both pro-inflammatory and anti-inflammatory, have a higher concentration when compared to healthy women.

There are reports that GDM increases the production of only inflammatory cytokines in the blood (16). However, some authors also showed an increase in both groups of cytokines from mothers with GDM (38). This dichotomy may be related to the organism’s attempt to promote balance between different cytokines and chemokines to favor homeostasis. According to Diaz et al., in the breast milk of healthy women, a higher concentration of anti-inflammatory cytokines is observed compared to the inflammatory ones (39).

Hormones and growth factors are also bioactive components present in human milk. Their effect varies between direct effects on breast and milk production, while others may contribute to the growth and differentiation of an infant ‘s tissue (6, 7, 22). The growth factors mainly influence the growth and development of the gastrointestinal tract but may affect glucose metabolism and newborn growth (26, 40). However, the differences in the presence of growth factors in the colostrum from mothers with gestational diseases are rare in the literature. In the colostrum from GDM mothers compared to healthy ones, we compared the concentration of VEGF, FGF-basic, GM-CSF, PDGF, and G-CSF and found a decreased concentration of GM-CSF, with fewer GDM mothers producing high levels of this compound. In contrast, the concentration of PDGF tended to be higher, with more GDM mothers producing a high level of the compound.

The colostrum contains GM-CSF responsible for regulating the proliferation, differentiation, and survival of milk monocytes (41). The monocytes in breast milk, indeed, are present at higher concentrations in the colostrum, and the decreased concentration of GM-CSF may also decrease the number of those cells. Regardless of PDGF, GM-CSF is produced in higher concentration for most GDM mothers. PDGF is a promoter of cell proliferation, its function in breast milk is not completely clear, and it may be linked to angiogenesis. Its concentration in colostrum sometimes varies from pre-term to term delivery (23, 42). As soon as it is a cell proliferation stimulus, when it is increased in the colostrum could induce wrong and uncontrolled cell proliferation.

A Spearman correlation analysis was performed to verify the association of the biomarkers present in the colostrum to variables such as, gestational age, weight, BMI and weight gain. After this analysis, it was possible to verify that the women with GDM had a strong positive correlation between biomarkers and numerical variables, such as weight, weight gain, and BMI. In the group of women at usual risk, the graph is more dispersed and with more correlated clusters among the biomarkers themselves. After interpreting the graphs, we found that in the GDM group, there is a positive correlation between cytokines in general, with a stronger correlation between inflammatory and anti-inflammatory cytokines.

Studies on colostrum immunology, especially in GDM, are still scarce in the literature. The studies that analyzed the immune profile in women with GDM mainly evaluated women’s blood and not their milk. The data presented in general corroborate the findings of Fujimori et al. (2017), who support the hypothesis that the changes in the organism induced by GDM can alter the immune composition of the colostrum (11).

The present study has some limitations: it had a small sample size, it only had one breast milk collection point, and the diagnosis of diabetes was from medical records. However, it is a critical study, and the main strength is the evaluation of several parameters in the colostrum and the analysis of bioactive compounds in the colostrum of women with gestational diabetes. Therefore, the study used a rigorous and detailed statistical analysis and brought new and valid results.

## Conclusion

The composition of the colostrum is modified in mothers with GDM, and certain cytokines and growth factors have altered its concentration, in comparison with healthy women. New studies could be performed to determine the impact of the alteration in the concentration of those bio-compounds on newborns’ health throughout their life.

## Data Availability Statement

The original contributions presented in the study are included in the article/**Supplementary Material**. Further inquiries can be directed to the corresponding author.

## Ethics Statement

The studies involving human participants were reviewed and approved by Comitê de Ética em Pesquisa do Hospital Risoleta Tolentino Neves. The patients/participants provided their written informed consent to participate in this study.

## Author Contributions

ACSA: collected, processed, and analyzed samples. MNO: collected and processed samples. RCVS: performed statistical analysis. FC: developed the Spearman correlation. ES and AT: contributed to Luminex assay. AMCF: provided the Luminex kit. GS-N: helped with the analysis, especially with radar analysis. TUM: idealized the project, wrote the manuscript, and supervised all phases. All authors contributed to the article and approved the submitted version.

## Funding

This project did not have funding. All the measurements were developed with the kit provided for another project grant. Thus, the indirect cost came from Fundação de Amparo a Pesquisa de Minas Gerais (FAPEMIG), Conselho Nacional de Ciência e Tecnologia (CNPq). and Pró-Reitoria de Pesquisa da Universidade Federal de Minas Gerais (PRPq-UFMG). AF and TM are research fellows from CNPq (TM research scholarship # 308877/2018-7).

## Conflict of Interest

The authors declare that the research was conducted in the absence of any commercial or financial relationships that could be construed as a potential conflict of interest.

## Publisher’s Note

All claims expressed in this article are solely those of the authors and do not necessarily represent those of their affiliated organizations, or those of the publisher, the editors and the reviewers. Any product that may be evaluated in this article, or claim that may be made by its manufacturer, is not guaranteed or endorsed by the publisher.
