# Supplementary material for: Gestational Diabetes Mellitus Changes Human Colostrum Immune Composition
Source: Front Immunol. 2022 Jun 20;13:910807. doi: 10.3389/fimmu.2022.910807 (PMC9251182; doi:10.3389/fimmu.2022.910807)
Supplement: Supplementary file 1 [file Table_1.docx]

Supplementary Table:

| **USUAL RISK** | | | | | | | | | | **GDM** | | | | | | | | **Valor p^a^** |
| --- | --- | --- | --- | --- | --- | --- | --- | --- | --- | --- | --- | --- | --- | --- | --- | --- | --- | --- |
|  | | | | | | | | | |  |  |  |  |  |  |  |  |  |
| **Cytokines** | | **No. of detectable samples** | | **Median (pg/mL)** | | **Minimum**  **(pg/mL)** | | **Maximum**  **(pg/mL)** | | **No. of detectable samples** | | **Median (pg/mL)** | | **Minimum**  **(pg/mL)** | | **Maximum**  **(pg/mL)** | |  |
|  | |  | |  | |  | |  | |  | |  | |  | |  | |  |
| **CCL11** | | 14 | | 3,6 | | 0,4 | | 149,8 | | 11 | | 9,4 | | 0,6 | | 109,1 | | 0,29 |
| **IL-2** | | 14 | | 1,4 | | 0,9 | | 8,9 | | 7 | | 3,2 | | 2,6 | | 15,2 | | 0,85 |
| **IL-4** | | 9 | | 0,2 | | 0,2 | | 0,5 | | 10 | | 0,3 | | 0,1 | | 2,2 | | 0,07 |
| **IL-5** | | 14 | | (18,79±6,57)* | |  | |  | | 11 | | (17,96±9,60)* | |  | |  | | 0,80ᶿ |
| **IL-7** | | 7 | | 14,6 | | 29,2 | | 50,2 | | 7 | | 2,8 | | 2,2 | | 41,7 | | 0,55 |
| **IL-9** | | 5 | | 0 | | 2144,6 | | 2919,0 | | 7 | | 9,5 | | 8,6 | | 2558,3 | | 0,60 |
| **IL-10** | | 2 | | 0 | | 3,5 | | 4,8 | | 7 | | 0,7 | | 0,7 | | 9,1 | | 0,03 |
| **IL-13** | | 1 | | 0 | | 0,1 | | 0,1 | | 4 | | 0 | | 0,1 | | 0,6 | | 0,06 |
| **IL-1 ra** | | 14 | | 529,7 | | 135,9 | | 749,1 | | 5 | | 0 | | 200,5 | | 641,9 | | 0,01 |
| **CXCL10** | | 7 | | 29,1 | | 58,2 | | 3486,8 | | 7 | | 312,9 | | 238,4 | | 32992,8 | | 0,30 |
| **CCL2** | | 6 | | 0 | | 3,8 | | 2609,7 | | 5 | | 0 | | 11,6 | | 775,7 | | 0,90 |
| **CCL3** | | 14 | | 75,0 | | 2,1 | | 1504,4 | | 11 | | 43,4 | | 1,7 | | 3997,7 | | 0,91 |
| **CCL4** | | 13 | | 118,9 | | 14,9 | | 306,7 | | 11 | | 225,8 | | 13,7 | | 2002,5 | | 0,06 |
| **CCL5** | | 12 | | 8,4 | | 2,1 | | 249,5 | | 9 | | 30,6 | | 6,4 | | 173,2 | | 0,35 |
| **TNF-α** | | 11 | | (25,17±20,95)* | |  | |  | | 10 | | (32,67±28,80)* | |  | |  | | 0,46ᶿ |
| **IFN-γ** | | 14 | | 6,4 | | 0,1 | | 10,4 | | 10 | | 11,0 | | 1,9 | | 82,2 | | 0,04 |
| **IL-1β** | | 14 | | 1,6 | | 0 | | 87,5 | | 11 | | 0,6 | | 0,6 | | 19,8 | | 0,70 |
| **IL-6** | | 9 | | 1,8 | | 0,7 | | 6,7 | | 11 | | 11,6 | | 1,6 | | 64,8 | | 0,01 |
| **IL-8** | | 6 | | 0 | | 5,5 | | 7191,8 | | 5 | | 0 | | 10,9 | | 1507,8 | | 0,99 |
| **IL-12** | | 0 | |  | |  | |  | | 3 | | 0 | | 0,4 | | 0,7 | | 0,04 |
| **IL-15** | | 8 | | 35,4 | | 35,4 | | 97,4 | | 10 | | 86,3 | | 35,4 | | 215,4 | | 0,01 |
| **IL-17** | | 14 | | 18,9 | | 15,2 | | 26,9 | | 11 | | 17,9 | | 4,3 | | 29,8 | | 0,70 |
|  |  | |  | |  | |  | |  | |  | |  | |  | |  |  |
| **VEGF** | | 14 | | 4947 | | 992,2 | | 114166 | | 10 | | 5468 | | 0 | | 28564 | | 0,72 |
| **FGF** | | 14 | | 4,33 | | 3,69 | | 12,52 | | 9 | | 5,99 | | 0 | | 40 | | 0,36 |
| **GM-CSF** | | 14 | | 0,46 | | 0,32 | | 24,71 | | 5 | | 0 | | 0 | | 0,6 | | 0,0004 |
| **PDGF** | | 14 | | 18,87 | | 11,1 | | 77,41 | | 11 | | 28,97 | | 13,31 | | 108,3 | | 0,055 |
| **GCSF** | | 14 | | 143,9 | | 35,6 | | 203 | | 11 | | 165 | | 59,1 | | 1014 | | 0,19 |
|  | |  | |  | |  | |  | |  | |  | |  | |  | |  |
|  | |  | |  | |  | |  | |  | |  | |  | |  | |  |
